# Psychopathological profiles of inmates convicted for femicide, other familial homicides, or attempted homicides

**DOI:** 10.1111/1556-4029.70310

**Published:** 2026-03-09

**Authors:** Biancamaria Treves, Carmen Santoriello, Gaia De Angelis, Carmela Di Filippo, Fabio del Duca, Ludovica Caprio, Gaetana Termoli, Paola Frati, Aniello Maiese, Antonio Maria Pagano

**Affiliations:** ^1^ Department of Anatomical, Histological, Forensic and Orthopedic Sciences Sapienza University of Rome Rome Italy; ^2^ Unità Operativa Complessa U.O.C. Department of Adults and Minors Healthcare Criminal Area, Local Health Authority of Salerno Salerno Italy; ^3^ Department of Occupational Medicine University of Rome Tor Vergata Rome Italy

**Keywords:** familial homicide, femicide, homicide, intimate partner homicide, psychopathy

## Abstract

Femicides and family‐related homicides represent a significant concern, with over 50% of homicides occurring within the family setting. This study aimed to explore the complex factors, including psychological aspects, behind such crimes, focusing on a sample of inmates incarcerated for homicide, attempted homicide, or femicide. The study analyzed data from 11 inmates (8 males, 3 females) incarcerated for homicide or attempted homicide. Demographic, clinical, and criminological data were collected, and psychological functioning was assessed using the Brief Psychiatric Rating Scale (BPRS) and the Symptom Checklist‐90‐Revised (SCL‐90‐R). The sample had an average age of 48 years and generally low educational attainment. Significant mental health issues were identified, with 45.5% of participants reporting a history of substance dependence and suicide attempts. Psychological assessments revealed a predominance of anxiety and depressive symptoms, particularly within the domain of negative affectivity. Findings suggest a link between emotional and relational dysfunction and violent behavior, indicating the need for targeted psychological interventions. However, given the small sample size, these results should be interpreted with caution and considered preliminary. Future research should employ more advanced statistical methods and include victims' perspectives to better identify risk factors and improve prevention strategies.

**Highlights**

- Over 50% of homicides occurr within a family context.
- Psychological tests revealed high levels of anxiety and depressive symptoms.
- 45.5% of participants had a history of substance abuse and suicide attempts.
- Emotional and relational dysfunctions emerged as key factors in violent behavior.
- Findings highlight the need for targeted psychological interventions in prisons.

## 1 INTRODUCTION

In recent years, crimes committed within the family sphere, particularly femicides, have garnered increasing attention from both society and the scientific community, constituting a significant proportion of total homicides in Italy. As a preliminary clarification, femicide can be defined as “the killing of a woman because she is a woman” [1, 2].

According to the 2024 report by the Italian Ministry of the Interior, 321 homicides were recorded, of which 153 occurred within the family context. Among these, 113 victims were women, and 61 were killed by a partner or ex‐partner [3].

International data reveal a similar pattern. In 2022, approximately 48,800 women and girls worldwide were killed by intimate partners or family members—over 130 victims each day [4]. Although overall homicide rates have declined globally, gender‐related killings have not followed the same trend, highlighting their persistence as a distinct form of violence driven by relational and social dynamics. Although most homicides worldwide involve men (80% in 2022), women are the primary victims of domestic homicides: they represent about 53% of all family‐related victims and 66% of those killed by a partner.

Familial homicides, particularly femicides, represent a complex phenomenon influenced by multiple factors. The scientific literature has examined the psychopathological determinants of these crimes, seeking to understand the extent to which psychiatric disorders may constitute a risk factor for partner or familial homicide. However, the causal link between mental illness and homicidal behavior remains debated. Instead, dysfunctional relational dynamics and socio‐cultural factors often play a crucial role, rather than by a diagnosed psychiatric disorder alone.

Given the relevance of this issue, it is essential to investigate not only psychiatric disorders but also individual responses to distress, stress, and frustration within a societal context marked by the decline of traditional mediating roles, such as those played by families and institutions.

Within this context, the present exploratory study seeks to contribute new empirical evidence from the Italian correctional system—an area still underrepresented in international literature. Specifically, it examines the demographic, clinical, and psychological characteristics of 11 inmates convicted of homicide, attempted homicide, or femicide against family members or intimate partners. By focusing on emotional functioning and psychopathological profiles, this study aims to shed light on the psychological mechanisms underlying extreme family violence and to inform future prevention and rehabilitation strategies.

## 2 MATERIALS AND METHODS

### 2.1 Population of the study

This study analyzed anonymized data from 11 inmates incarcerated for homicide or attempted homicide, with victims belonging to their family or close emotional circle. Data were collected at the “Antonio Caputo” Prison in Salerno, Italy, during a 5‐year period of time (between November 2019 and November 2024). All participants were included in the study after providing written informed consent.

Upon admission to prison, inmates were assessed within 24 h by a healthcare team comprising a psychiatrist and a psychologist to evaluate suicide risk, clinical status, and any signs of psychological distress.

### 2.2 Demographic and psychiatric evaluation

The variables evaluated included demographic information (gender, age, education level, marital status, and employment), clinical data (history of suicide attempts, presence of substance use disorders, and psychiatric diagnoses), and criminological data (type of offense and relationship with the victim).

To achieve a comprehensive understanding of the inmates' psychological functioning, two complementary assessment tools were employed: the Brief Psychiatric Rating Scale (BPRS 4.0; [5]) and the Symptom Checklist‐90‐Revised (SCL‐90‐R; [6]). The rationale for using both instruments lies in their different yet synergistic perspectives: the BPRS, a clinician‐administered scale, captures observable psychopathological symptoms and behavioral manifestations through structured clinical evaluation, whereas the SCL‐90‐R, a self‐report questionnaire, provides insight into the inmates' subjective experience of psychological distress. The integration of clinician‐rated and self‐reported measures allows for a more balanced and multidimensional assessment, reducing potential biases inherent in relying on a single evaluation method and enhancing diagnostic accuracy.

The BPRS [5] administered by a psychiatrist, is a semi‐structured interview format designed to assess the presence, severity, and clinical course of a wide range of psychopathological symptoms. It is considered a reliable and versatile tool, useful for monitoring symptom changes over time and supporting clinical decision‐making across various settings.

The scale consists of 24 items evaluating aspects related to behavior, mood, thought processes, perceptions, and social functioning. Each item is rated on a scale from 1 to 7 (1 = absence of symptoms; 7 = very severe symptoms). The final score provides an overall measure of psychopathological severity, with a cut‐off value of 41 indicating significant psychological impairment.

Furthermore, based on an analysis conducted on 250 psychiatric patients, [7] this score allows for the identification of five distinct clusters:

- Disorganization (including the following items: “bizarre behavior,” “self‐neglect,” “disorientation,” and “conceptual disorganization”), with a range from 4 to 28;
- Negative affectivity (including “somatic concerns,” “anxiety,” “depression,” “suicidal risk,” “guilt feelings,” and “distractibility”), ranging from 6 to 42;
- Positive symptoms (including “unusual thought content,” “hallucinations,” “hostility,” and “suspiciousness”), ranging from 4 to 28;
- Expanded affectivity (including “elevated mood,” “grandiosity,” “excitement,” and “motor hyperactivity”), ranging from 4 to 28;
- Negative symptoms (including “blunted affect,” “emotional withdrawal,” and “motor retardation”), with a range from 3 to 21.

The Symptom Checklist‐90‐Revised [6] is a self‐report questionnaire that measures the severity of psychological distress experienced by the individual in the week preceding the assessment. The Italian version, validated with excellent psychometric properties, presents reliability values (Cronbach's *α*) ranging from 0.68 to 0.87 for the nine explored symptom dimensions and 0.97 for the global index (Global Severity Index—GSI).

The scale comprises 90 items designed to evaluate nine distinct psychopathological dimensions: Somatization, Obsessive‐compulsiveness, Interpersonal sensitivity, Depression, Anxiety, Hostility, Phobic anxiety, Paranoid ideation, Psychoticism. Each symptom is rated on a scale from 0 to 4 (0 indicating no distress and 4 representing extreme severity).

In addition to these dimensions, the SCL‐90‐R provides three global indices: (1) GSI, which measures overall psychological distress; (2) Positive Symptom Total (PST), indicating the total number of reported symptoms; (3) Positive Symptom Distress Index (PSDI), assessing the average intensity of symptoms.

SCL‐90‐R scores are standardized, with a mean of 50 and a standard deviation of 10. Gender‐specific norms allow comparisons using T‐scores and percentiles, offering a detailed assessment of individual psychopathological profiles.

### 2.3 Data analysis

The statistical analysis was primarily descriptive, involving the estimation of means, standard deviations, and ranges for the variables under consideration. This methodological approach facilitated a comprehensive characterization of the subjects in relation to the measured factors, encompassing socio‐demographic, clinical, criminological variables, psychopathological symptoms (BPRS) and perceived psychological distress (SCL‐90‐R). Given the small sample size (11 subjects), the results were interpreted with caution, prioritizing the description of observed trends rather than the generalization of findings.

The data processing complied with the general authorization for scientific research purposes granted by the Italian Data Protection Authority (March 1, 2012, as published in Italy's Official Journal no. 72 dated March 26, 2012), as the data were fully anonymized and did not entail any significant personalized impact on the data subjects. Written informed consent was obtained from all participants. In accordance with Italian regulations, formal approval by an institutional and/or licensing ethics committee was not required because the study was observational in nature, involved no experimental or interventional protocols, and was based exclusively on anonymized data. All procedures, including assessment protocols and screening, were conducted in line with World Health Organization recommendations and in conformity with the ethical guidelines of the Declaration of Helsinki (1975).

## 3 RESULTS

### 3.1 Socio‐demographic, clinical, and criminological characteristics

The general characteristics of the sample are presented in Table 1.

**TABLE 1:** Demographic, clinical, and criminological characteristics of the sample.

Subject characteristics		Total sample (*n* = 11)
		Frequencies (%)
Sex	Male	8 (72.7%)
	Female	3 (27.3%)
Age	<40	3 (27.3%)
	≥40	8 (72.7%)
Educational level	Elementary license	4 (36.4%)
	Lower secondary school qualification	6 (54.5%)
	High school diploma	1 (9.1%)
Marital status	Unmarried	5 (45.5%)
	Married	4 (36.4%)
	Separated/divorced	2 (18.2%)
Employment status	Unemployed	6 (54.5%)
	Employed	2 (18.2%)
	Retired	3 (27.3%)
Previous suicide attempts	No	6 (54.5%)
	Yes	5 (45.5%)
Pathological addiction	No	6 (54.5%)
	Yes	5 (45.5%)
Diagnosis	Personality disorder	2 (18.2%)
	Schizophrenia spectrum disorder	1 (9.1%)
	Mood disorder	2 (18.2%)
	Narcissistic personality traits	2 (18.2%)
	Absence of psychopathology	4 (36.3%)
Offense	Murder	6 (54.5%)
	Attempted murder	5 (45.5%)
Offense victim	Former partner	9 (81.8%)
	Daughter	1 (9.1%)
	Father	1 (9.1%)

The sample of this study consists of Italian 11 inmates, including 8 are males and 3 females. The average age at the time of the crime was 48 ± 17.01 years old (range 22–75). Considering genders separately, the average age of the females was 44.67 years ± 23.01 (range 22–68), while the average age of the males is 49.25 years ± 15.98 (range 26–75).

About educational level, the majority of the inmates completed lower secondary school (54.5%), followed by 36.4% with only primary school education and just 9.1% with a high school diploma. Only 18.2% of inmates were employed, while the larger portion of the inmates were unemployed (54.5%), and the rest were retired.

In terms of marital status, the majority of inmates was not married. About mental health history, 45.5% of the inmates reported a previous suicide attempt. Additionally, 45.5% of the inmates had a substance use disorder (drugs or alcohol), whereas the rest did not show any form of dependency.

Regarding the psychological aspect, the following disorders were diagnosed: schizophrenia spectrum disorder (1 case), personality disorder (2 cases), mood disorder (2 cases), narcissistic personality traits (2 cases). In four cases, no psychopathology was identified (36.4%).

Concerning the offenses committed, 45.5% of the inmates attempted homicide, while 54.5% committed homicide. The victims were primarily ex‐partners (81.8%), followed by a daughter and a father.

### 3.2 Psychopathological profile—BPRS scale results

The analysis of the scores of the BPRS revealed that only one subject reached the clinical cut‐off score of 41, indicating the presence of a psychopathological condition. The overall average score for the sample was 34 (SD = 4.67; range = 27–41), suggesting moderate symptom intensity in most of the inmates.

**FIGURE 1:**
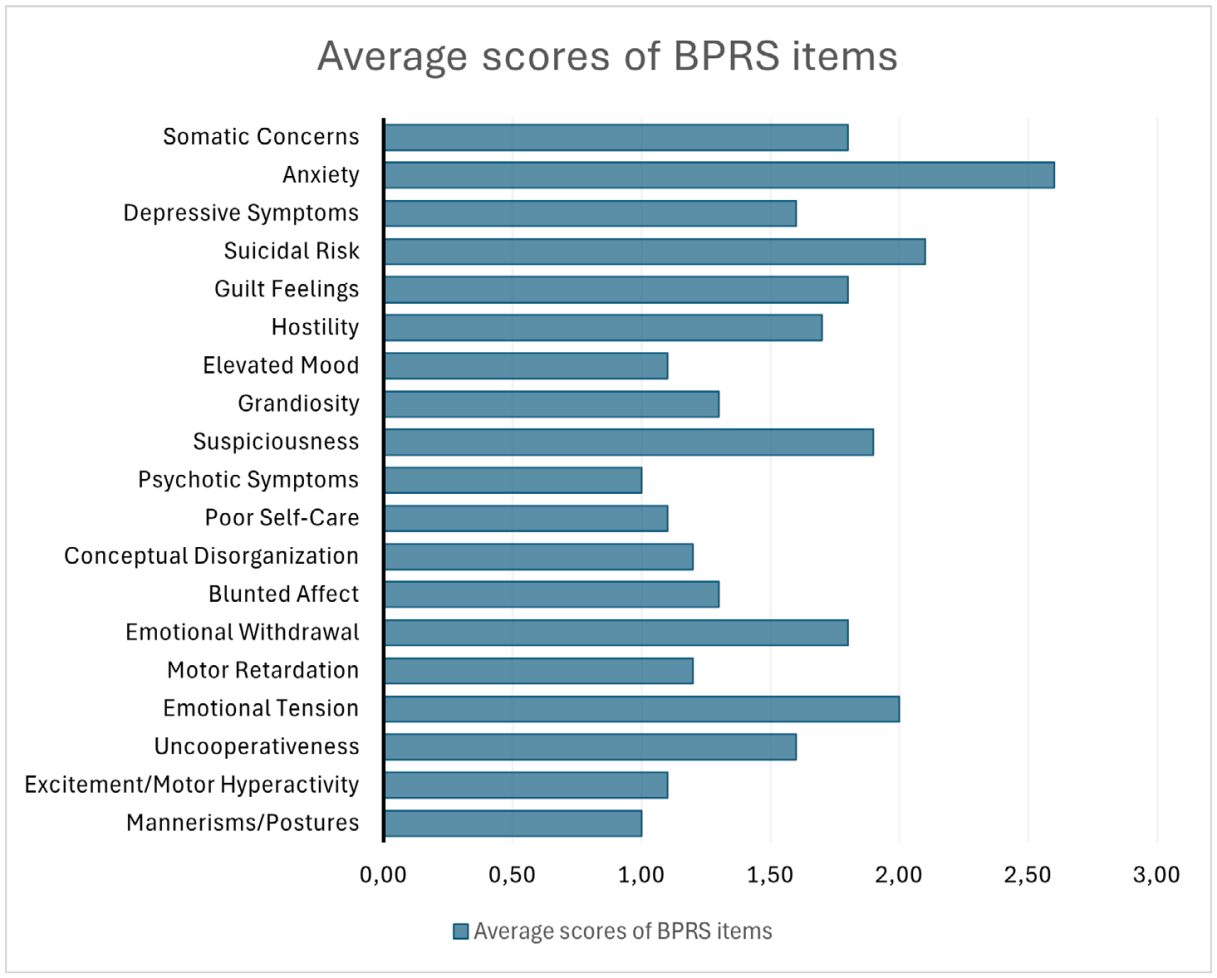
Average scores of the sample for each item of the Brief Psychiatric Rating Scale (BPRS).

The average scores for each interview item are presented in Figure 1, with higher scores observed in anxiety and suicide risk.

**FIGURE 2:**
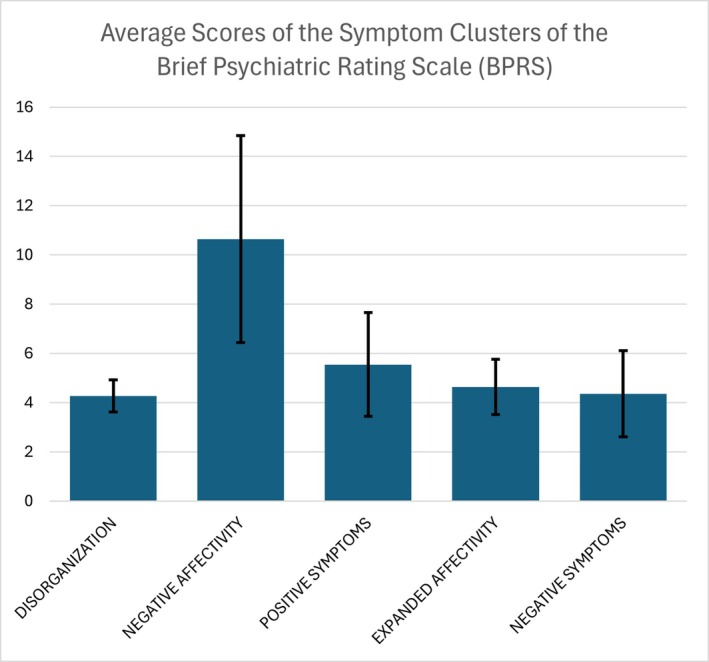
Average scores of the symptom clusters of the Brief Psychiatric Rating Scale (BPRS).

Analysis of the symptom clusters revealed that “negative affectivity” recorded the highest average score (M = 10.64; SD = 4.20; range = 6–19), with considerable variability, ranging from moderate to higher levels (Figure 2). This indicates a component of anxiety and depression in the sample, associated with moderate suicide risk and feelings of guilt.

The “positive symptoms” cluster recorded an average score of 5.55 (SD = 2.11; range = 4–10), suggesting the presence of mild symptoms such as suspicion and hostility, but not reaching the severity typical of psychotic disorders. The scores were variable, with some inmates reporting more marked symptoms than others, but without signs of severe psychopathology.

The “expanded affectivity” cluster showed low average scores (M = 4.64; SD = 1.12; range = 4–7), indicating a low prevalence of symptoms related to elevated mood, grandiosity, or excitement. Although occasional manifestations of expanded affectivity were observed, they did not significantly impact on the overall clinical picture.

The “negative symptoms” cluster recorded an average of 4.36 (SD = 1.75; range = 3–8), reflecting moderate symptoms of flattened affect and emotional withdrawal. These scores suggest emotional and relational difficulties, but without signs of extreme isolation.

The “disorganization” cluster showed the lowest average score (M = 4.27; SD = 0.65; range = 4–6), suggesting that the inmates do not exhibit clear signs of disorientation, bizarre behavior, or conceptual disorganization.

In summary, the clinical profile of the inmates primarily highlights symptoms of anxiety, motor tension, and negative affectivity, with mild levels of suspicion and hostility, and no signs of severe psychopathology. The profile delineates a condition of moderate psychological distress, characterized by an anxious‐depressive component and a predominance of emotional and relational difficulties.

### 3.3 Perceived psychological distress—Symptom checklist‐90‐revised results

The results obtained from the administration of the Symptom Checklist‐90‐Revised (SCL‐90‐R) do not indicate significant deviations from the normative reference sample for the GSI (M = 45.14; SD = 5.42; range = 37–55). This suggests that, overall, the considered population of inmates do not report particularly high levels of perceived psychological distress compared to the general population.

Regarding the PST, which represents the total number of reported symptoms regardless of distress intensity, the mean score observed was 46.19 (SD = 6.66; range = 35–57), also aligning with values found in general population. This indicates that, although the variety and breadth of reported symptoms are relatively wide, they do not significantly deviate from the expected range.

The PSDI, which measures the average severity of perceived psychological distress in relation to the reported symptoms, showed a mean score of 53.67 (SD = 9.40; range = 42–73). This index is particularly useful for interpreting individual responses, revealing whether distress has been amplified or minimized. The observed PSDI values fall within the average range of normative scores, suggesting that inmates do not tend to exaggerate or downplay their perceived psychological distress.

However, the mean distress intensity appears slightly higher than other indices, such as the GSI and PST, which could indicate that among the reported symptoms, those perceived as more distressing have a greater impact on the subjective experience of discomfort. This observation is consistent with the overall profile of the sample, where, despite the absence of clinically significant symptoms, some reported symptoms may reflect a deeper underlying psychological distress. Therefore, the PSDI suggests that in our population perceived distress related to reported symptoms may be influenced by individual or contextual factors, such as the condition of incarceration or the presence of ineffective coping strategies.

**FIGURE 3:**
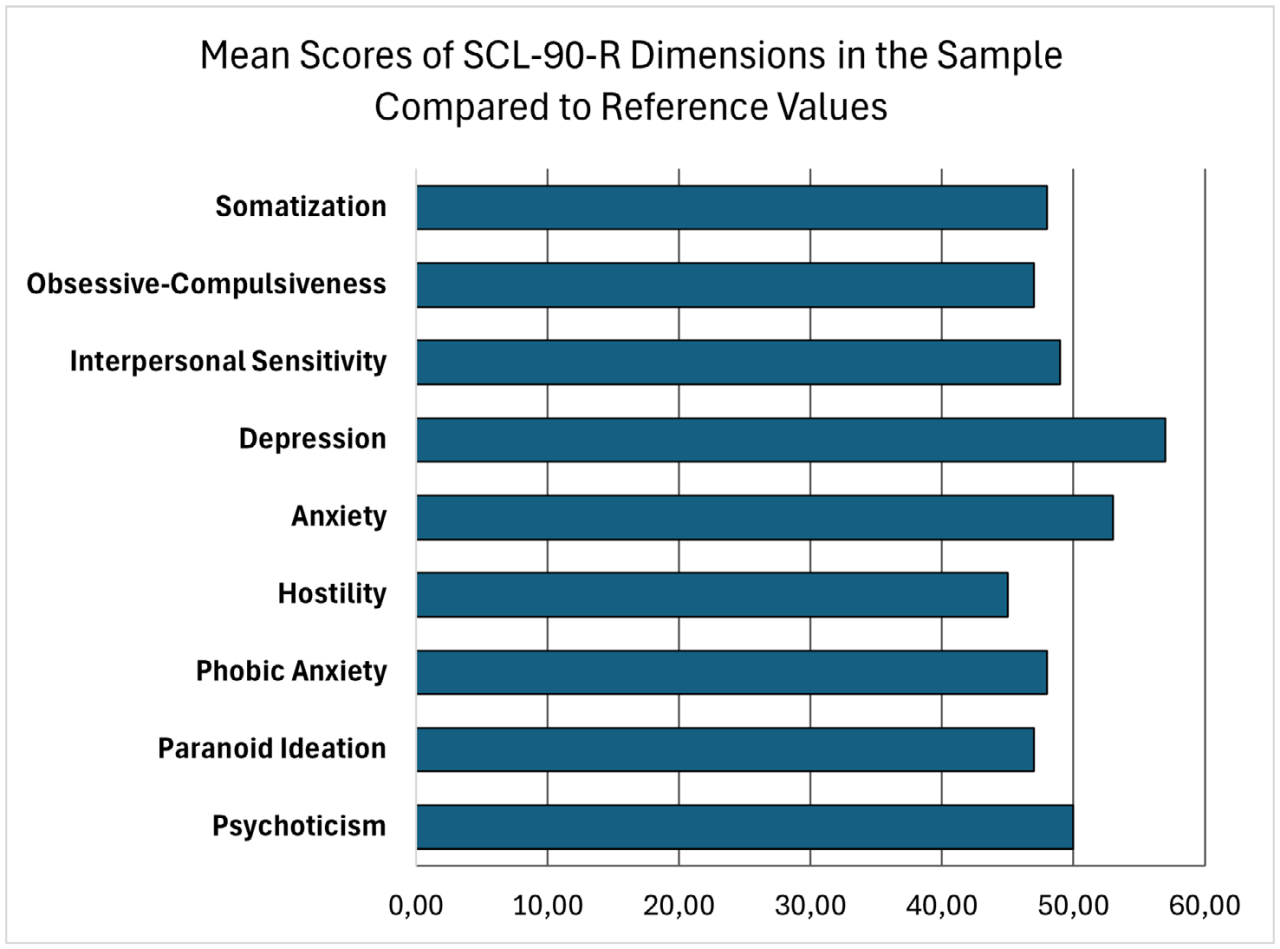
Mean scores of Symptom Checklist‐90‐Revised (SCL‐90‐R) dimensions in the sample compared to reference values.

Figure 3 shows the mean scores of the nine psychopathological dimensions assessed with the SCL‐90‐R. Somatization scores fall within normative limits, with a medium score of 47.73 (SD = 6.32; range = 41–59). This indicates a moderate perception of bodily symptoms without excessive amplification of somatic distress. Similarly, the obsessive‐compulsive dimension (M = 46.99; SD = 7.26; range = 41–64) reflects a limited presence of intrusive thoughts or repetitive behaviors. Interpersonal sensitivity has a mean score of 49.54 (SD = 10.29; range = 43–77), suggesting that some inmates experience difficulties in social relationships, such as feelings of inadequacy or discomfort in interactions with others. Notably, the scores for depression (M = 56.59; SD = 15.33; range = 44–94) and anxiety (M = 52.95; SD = 16.28; range = 42–96) resulted significantly higher than other dimensions, indicating intense emotional experiences, including deep sadness, loss of interest, guilt, and hyperarousal. Conversely, the hostility (M = 44.78; SD = 2.86; range = 43–51) and paranoid ideation (M = 46.75; SD = 3.70; range = 42–54) dimensions show lower mean scores, suggesting a reduced incidence of aggression, irritability, or excessive suspiciousness. The phobic anxiety dimension (M = 47.90; SD = 4.31; range = 46–60) indicates mild symptoms without clinically significant manifestations. Finally, the psychoticism dimension presents a mean score of 50.58 (SD = 8.65; range = 44–68), which remains within normative limits, but suggests a slight tendency toward manifestations such as social withdrawal or unusual thoughts.

Given the limited sample size and the descriptive nature of the analyses, these findings should be interpreted as preliminary and exploratory, providing useful clinical insights but not allowing for generalizations to the broader population of inmates.

## 4 DISCUSSION

Our sample of inmates convicted for familial homicide and attempted homicide confirms a well‐established and widely documented trend in the literature: the male predominance among perpetrators of such crimes.

Indeed, a distinct feature of both the incarcerated population and homicide perpetrators is the predominance of male offenders. As of December 31, 2024, Italian prisons housed 61.861 inmates, including 51.963 men and 2.698 women with males comprising about 94.8% of the prison population. This distribution has remained stable over time [8]. Similarly, regarding homicide perpetrators, in 2022, a total of 334 homicides were recorded in Italy, with men responsible for 93.3% of these crimes [9].

ISTAT data from 2018 to 2019 shows a slightly different trend, with most individuals convicted of murder falling within the 18–44 age range. Among female offenders, 56.5% committed their crimes between the ages of 35 and 44, whereas for males, the most represented age group was 25–34, accounting for over one‐third (218 individuals) of male convictions [10].

The predominance of individuals over 40 years of age in our sample (72.7%) may suggest that perpetrators of familial crimes are generally adults with prior life and familial experiences, although this cannot be generalized beyond the current small sample.

Recent studies have highlighted that familial homicides, especially those committed within intimate relationships, are often linked to dynamics of jealousy, relational dissatisfaction, and difficulties in managing emotional conflicts [2].

The age differences between men and women in our sample (45 years for women and 49 for men) could reflect distinct psychological maturation trajectories and varying exposures to social and psychopathological risk factors, although caution is needed due to the small and unbalanced sample. Specifically, the lower average age of women could reflect earlier exposure to stressful life experiences.

Additionally, a particularly noteworthy finding is that 45.5% of inmates reported previous suicide attempts, which may be associated with impulsive behavioral tendencies and emotional dysregulation, potentially relevant for violent behaviors.

According to the study by Kraanen et al., conducted on a group of men and women undergoing treatment for substance dependence, substance use disorders emerged as significant predictors of any form of IPV, with approximately one‐third of substance users involved in IPV. In particular, the co‐occurrence of alcohol and cocaine use disorders—compared to alcohol abuse or dependence alone—was positively associated with an increased likelihood of IPV involvement. The authors emphasize that, although correlations exist between substance abuse and IPV, a direct causal relationship cannot be established. Factors such as personality traits and post‐traumatic stress disorder may increase the risk of both substance abuse and violent behavior [11, 12].

This aspect is particularly relevant in the context of familial crimes, where complex interpersonal dynamics and relational difficulties can be exacerbated by psychophysiological disturbances caused by dependencies.

Consistent with these findings, the study by Golinelli et al. indicated that the risk of IPV significantly increased with both personal and partner substance use. When analyzed separately, individual substance use (OR = 4.67, *p* < 0.001) and partner substance use (OR = 6.12, *p* < 0.001) were both strong predictors of IPV. Even when examined simultaneously, each remained an independent predictor—individual use (OR = 2.78, *p* = 0.004) and partner use (OR = 4.18, *p* < 0.001) [13].

Concerning the psychological evaluation, the distribution of scores on the BPRS and SCL‐90‐R scales provides significant insights into the psychological characteristics associated with intrafamilial violence. The psychological traits identified in our study suggest a profile of moderate psychological distress, with a predominance of anxious and depressive symptoms. The presence of motor tension and negative affectivity, which constitute central aspects of the clinical picture, is accompanied by tendencies toward suspiciousness and hostility. These traits suggest a propensity for conflictual dynamics and distrust in interpersonal relationships, highlighting the need for a more in‐depth analysis of the emotional and relational factors that fuel violent dynamics. Such characteristics could be linked to dysfunctional interaction patterns, where violence emerges as a response to frustration or perceived threat. However, it is worth noting that the results obtained from the two instruments appear partially divergent: while the BPRS indicates moderate levels of anxious‐depressive symptoms, the SCL‐90‐R does not reveal significant deviations from normative values. This discrepancy may reflect differences in the type of assessment (clinician‐administered versus self‐report) or in the inmates' level of awareness and self‐perception of psychological distress. Therefore, these findings should be interpreted cautiously and warrant further investigation to better understand the interplay between objective clinical evaluations and subjective experiences of distress in this population.

The estimated prevalence of psychopathy in the general population ranges from 1% to 3.5% [14], while rising among the prison population [15].

Evidence from the literature suggests that the psychiatric profiles most frequently linked to intimate partner violence include narcissistic traits, antisocial personality disorder, and borderline personality disorder [16].

Other psychological vulnerability factors, such as narcissistic traits, impulsivity, emotional dysregulation, and substance abuse, may contribute to the escalation of violence. However, due to the exploratory design, no causal or generalizable links between these traits and homicidal behavior can be established.

The estimated prevalence of psychopathy among intimate partner aggressors varies widely, ranging from 3.9% to 42%, depending on the cut‐off criteria used and the country in which the study was conducted [17, 18, 19]. Although numerous studies have investigated the relationship between intimate partner homicide or violence and psychopathy, their findings produced contradictory results.

The review conducted by Robertson et al., which included 43 studies and over 13,000 participants, highlighted that an insufficient number of studies provided adequate statistical data to generate a reliable quantitative summary of the relationship between psychopathy and IPV [20].

A meta‐analysis conducted by Fox and DeLisi identified a strong association between psychopathy and homicide, reporting a large effect size of 0.68, with even higher association in cases of sexual homicide (0.71) [21].

Swogger et al. conducted a study on a prison sample of 172 inmates and found that individuals who engaged in domestic violence exhibited higher scores on the psychopathy facet linked to affective deficits. However, their findings did not indicate a significant association between overall psychopathy levels and the likelihood of being an intimate partner aggressor [22]. Carman et al. conducted a study on mental disorders among male perpetrators of intimate partner femicide (IPF) and male‐to‐male homicide (MMH). Approximately one‐third of offenders, regardless of homicide type, had been diagnosed with a mental disorder excluding substance‐related disorders. Rates of major mental disorders were similarly low across groups, at 11% [23]. A Swedish study comparing the characteristics of femicide perpetrators (*n* = 164) with those of general murderers (*n* = 690) found that only 4% of femicide perpetrators met the diagnostic criteria for psychopathy, as assessed by the PCL:SV scale. The study concluded that the psychopathic population is not overrepresented among femicide perpetrators. In contrast, the most prevalent disorders and disturbances in the sample were depressive disorders [24]. A systematic review on homicide and suicide, with a focus on intrafamilial cases, found that nearly all studies (44 out of 49) identified depression as a relevant factor, present in 15% of perpetrators. The overall prevalence of any mental disorder was 19%, while the prevalence of psychosis or personality disorders was 1% each [25]. Similarly, an analysis of a Spanish sample of on intimate partner homicide perpetrators reported an average score of 14.4 on the Psychopathy Checklist‐Revised (PCL‐R 2), with only 13.4% of participants scoring ≥25 (the threshold typically used to classify individuals as psychopaths). These offenders exhibit a relatively less antisocial profile compared to other criminal populations, and when mental disturbances or disorders are present, they are predominantly characterized by depressive symptoms [16].

A study which was conducted in Canada examining cases of parricide during a 5‐year period, using archival data, police reports, and psychiatric records, identified a total of 64 (37 patricides, 27 matricides). Evidence of mental disturbance was found in approximately 65.5% of offenders, with psychotic disorders (particularly schizophrenia) being most common, followed by depression and substance abuse. Matricides were more often preceded by psychiatric contact (16.7% vs. 8.3%), though not statistically significant. Furthermore, severe psychopathology was frequently observed in double parricide cases [26].

Kauppi et al. conducted a retrospective study identifying 65 cases of filicide. A psychotic state was diagnosed in 51% of maternal perpetrators and 20% of paternal cases. Personality disorders (either as a primary diagnosis or comorbid condition), were the most frequently observed pathology in paternal perpetrators (67%), predominantly exhibiting borderline features. Depression was present in 32% of mothers and 7% of fathers, while substance abuse was identified in 3% of mothers and 47% of fathers [27]. A study of male prisoners in Turkey comparing psychological characteristics of men femicide perpetrators with those who did not engage in violence against women found no significant differences in psychopathology associated with femicide [28]. Nevertheless, it is important to underline that the presence of a psychiatric disorder does not per se entail diminished criminal responsibility, as the individual's capacity to understand and to will (mens rea) may remain intact at the time of the commission of the offense.

In summary, although based on a small and exploratory sample, our findings suggest that inmates convicted of familial homicide or attempted homicide exhibit an intersection of emotional and relational difficulties with violent behaviors, indicating the need for targeted intervention and a deeper understanding of the underlying psychological dynamics. However, due to the limited sample size, descriptive nature of the analysis, and potential variability in individual cases, the results should be interpreted cautiously. Further research with larger, more diverse populations and more rigorous methodologies is necessary to validate these observations, explore potential causal relationships, and clarify the extent to which these traits may generalize to broader offender populations.

## 5 LIMITATIONS

One of the primary limitations of this study is the small sample size, which restricts the statistical power of the analysis and the generalizability of the findings to broader populations. A larger sample would allow for more reliable estimates and a greater ability to detect potential patterns or associations that may not be evident in a smaller cohort. Moreover, the limited sample size may introduce biases related to the specific characteristics of the individuals included in the study, potentially affecting the representativeness of the results. This constraint underscores the need for future studies with larger and more diverse samples to ensure greater external validity.

Additionally, this study is purely descriptive and did not apply inferential statistical techniques, precluding hypothesis testing or causal conclusions. These methodological limitations should be considered when interpreting the findings.

## 6 FUTURE RESEARCH

Future research should incorporate advanced statistical methodologies, such as regression models, multivariate analyses, or machine learning approaches, to better identify significant associations and predictive factors related to intimate partner homicide.

To address the aforementioned limitations, future studies should aim to increase the sample size and comparing psychopathological profiles across offender groups. This approach would help elucidate whether specific psychiatric dimensions are uniquely associated with intimate partner homicide or if they are common across different forms of violent behavior.

Furthermore, in cases of attempted homicide, expanding the research to include both victims and the underlying homicidal dynamics would provide a more comprehensive perspective on risk factors and behavioral patterns. Examining the psychological profiles, situational triggers, and relational dynamics of both perpetrators and survivors could contribute to a more nuanced understanding of the factors that differentiate fatal from non‐fatal incidents of intimate partner violence. Such findings could have significant implications for both clinical risk assessments and the development of targeted prevention and intervention strategies.

## 7 CONCLUSIONS

The present study investigated the psychopathological profiles of inmates convicted of familial homicide or attempted homicide, revealing a predominance of anxious‐depressive and negative affectivity‐related symptoms without severe psychopathology. Given the small and exploratory sample, these findings should be interpreted cautiously. Our results suggest a potential link between emotional and relational difficulties and violent behavior, highlighting the importance of targeted psychological assessment and intervention, but no causal claims can be made. This study provides preliminary insights and lays the groundwork for future research with larger samples and advanced analytical methods to better understand the psychological dynamics of familial and intimate partner homicide. Ultimately, such insights may contribute to the development of effective prevention and intervention strategies.

## CONFLICT OF INTEREST STATEMENT

The authors declare no conflict of interest.

## ETHICS STATEMENT

All procedures performed in studies involving human participants were in accordance with the ethical standards of the institutional and/or national research committee and with the 1964 Helsinki Declaration and its later amendments or comparable ethical standards.

## INSTITUTIONAL REVIEW BOARD STATEMENT

The data processing complied with the general authorization for scientific research purposes granted by the Italian Data Protection Authority (March 1, 2012 as published in Italy's Official Journal no. 72 dated March 26, 2012) since the data do not entail any significant personalized impact on the data subjects. Approval by an institutional and/or licensing committee was not required since experimental protocols were not applied in the study.

## INFORMED CONSENT STATEMENT

Informed consent was obtained from all subjects involved in the study.

## Data Availability

Research data are not shared.
